# Ileal Pouch Cancer Detected More than 30 Years after Restorative Proctocolectomy for Ulcerative Colitis

**DOI:** 10.70352/scrj.cr.25-0021

**Published:** 2025-03-13

**Authors:** Tetsuhiro Urashima, Kenji Tatsumi, Nao Obara, Eiichi Nakao, Sayumi Saito, Koki Goto, Hirosuke Kuroki, Kazutaka Koganei, Akira Sugita

**Affiliations:** Department of Inflammatory Bowel Disease (IBD), Yokohama Municipal Citizen’s Hospital, Yokohama, Kanagawa, Japan

**Keywords:** ulcerative colitis, restorative proctocolectomy, ileoanal pouch anastomosis, ileal pouch cancer, ileal pouch surveillance

## Abstract

**INTRODUCTION:**

The standard surgical treatment for ulcerative colitis (UC) is proctocolectomy with hand-sewn ileoanal pouch anastomosis (hand-sewn IPAA) or stapled ileal pouch anastomosis (stapled IPAA). The occurrence of cancer in the ileal pouch after surgery for UC is rare, and a consensus on surveillance for ileal pouch cancer has not been reached. We report a case of ileal pouch cancer diagnosed by pouchoscopy 33 years after restorative proctocolectomy with IPAA for UC.

**CASE PRESENTATION:**

A middle-aged man presented with positive fecal occult blood. The patient had undergone restorative proctocolectomy with IPAA for UC 33 years ago. Pouchoscopy had been performed every 2–3 years in the last 10 years. In April a year ago, he tested positive for fecal occult blood, and pouchoscopy revealed an ulcerative lesion and flat elevation in the ileal pouch on the proximal side of the ileoanal anastomosis. Targeted biopsies of the ulcerative lesion revealed low-grade dysplasia (LGD). After 4 months, pouchoscopy also showed an increase in the size of the flat elevation, but targeted biopsies of this lesion also showed LGD. One year later in August, endoscopic examination for hematochezia showed a full circumferential raised lesion with a white coat and mucus draining from a fistula near the anastomosis at the same site. Pathological examination identified adenocarcinoma in the ileal mucosa. The preoperative diagnosis was ileal pouch cancer after restorative proctocolectomy with IPAA for UC, cT4bN2M0 stage IIIB (UICC-TNM, 8th), and he underwent excision of the ileal pouch body and the ileoanal anastomosis. Pathological examination showed mucinous carcinoma in the ileal mucosa with chronic inflammation. The postoperative stage was pT3N0M0 stage IIA; no postoperative chemotherapy was administered, and at 6 months postoperatively, the patient remained recurrence free.

**CONCLUSION:**

Although ileal pouch cancer is rare, it can occur after a long period following ileal pouch surgery for UC. Endoscopic surveillance for ileal pouch cancer should be performed for early diagnosis and radical resection, especially if ileal pouch cancer occurs more than 10 years after the onset of UC.

## Abbreviations

ATZ
: anal transitional zone
IPAA
: ileal pouch anastomosis
LGD
: low-grade dysplasia
PSC
: primary sclerosing cholangitis
UC
: ulcerative colitis

## INTRODUCTION

The current standard surgical procedures for ulcerative colitis (UC) are restorative proctocolectomy with hand-sewn ileal pouch anastomosis (hand-sewn IPAA) or stapled ileal pouch anastomosis (stapled IPAA). The cumulative incidence of anal transitional zone (ATZ)/ileal pouch cancer is reported to be 0.9% at 5 years, 1.3% at 10 years, and 5.1% at 25 years after surgery,^1)^ and cancer arising in the ileal pouch after surgery for UC is rare. Therefore, there is no established consensus regarding surveillance for ileal pouch cancer, and guidelines differ between countries.^2–4)^ We report a case of ileal pouch cancer diagnosed by endoscopy 33 years after restorative proctocolectomy with IPAA for UC.

## CASE PRESENTATION

**Fig. 1:**
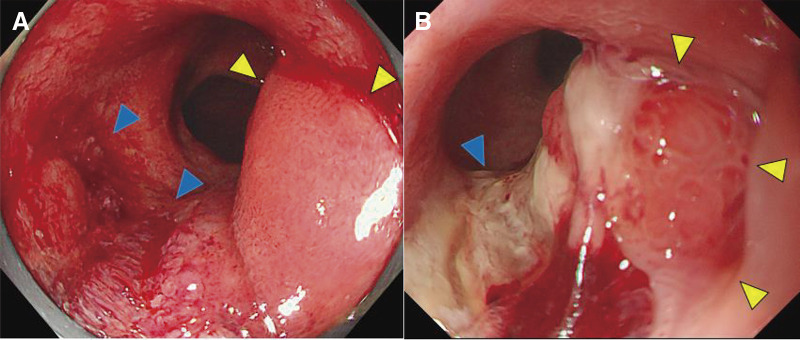
Ileal pouchoscopy in X-1. (**A**) The ulcerative lesion (blue arrow) and the flat elevation (yellow arrow) in the ileal pouch on the proximal side of the ileoanal anastomosis in April, X-1. (**B**) The increase in the elevation size (yellow arrow) and the ulcerative lesion (blue arrow) were observed in August, X-1.

**Fig. 2:**
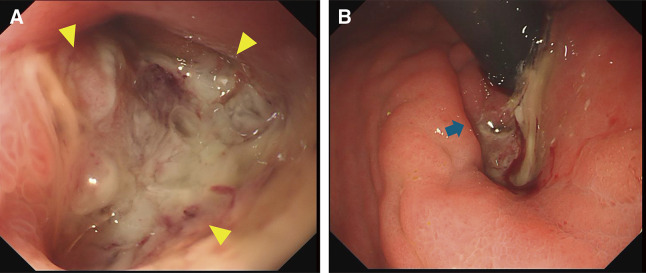
Ileal pouchoscopy in X. (**A**) The full circumferential raised lesion with a white coat on the proximal side of the ileoanal anastomosis (yellow arrow). (**B**) Mucus draining from the fistula near the anastomosis (blue arrow).

A middle-aged man presented with positive fecal occult blood. The patient had been diagnosed with UC and had undergone restorative proctocolectomy with IPAA for UC 33 years ago. After the surgery, pouchoscopy had been performed every 2 to 3 years in the last 9 years. In April a year ago, he tested positive for fecal occult blood, and pouchoscopy revealed an ulcerative lesion and a flat elevation in the ileal pouch on the proximal side of the ileoanal anastomosis. Targeted biopsies of the ulcerative lesion showed low-grade dysplasia (LGD). After 4 months, another pouchoscopy showed an increase in size of the elevation, but targeted biopsies of this lesion also showed LGD (**Fig. 1A** and **1B**). One year later in August, pouchoscopy for hematochezia showed the full circumferential raised lesion with a white coating and mucus draining from a fistula near the anastomosis at the same site. Pathological examination identified an adenocarcinoma in the ileal mucosa (**Fig. 2A** and **2B**). He was referred to our hospital for surgery.

**Fig. 3:**
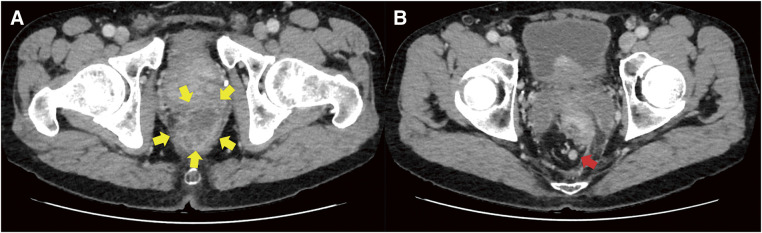
Enhanced CT. (**A**) A 48-mm mass with contrast enhancement was shown on the oral side of the ileoanal anastomosis in the ileal pouch (yellow arrow). (**B**) Enlarged lymph nodes were detected in the ileal mesentery (red arrow). CT, computed tomography

**Fig. 4:**
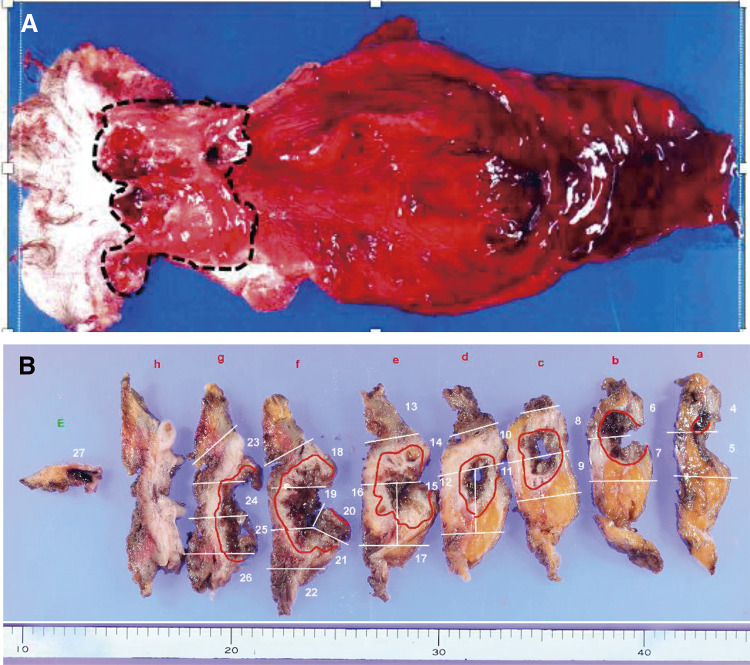
Pathological specimen. (**A**) The full circumferential type 5 tumor was shown on the proximal side of the ileoanal anastomosis (black dotted line). (**B**) Deep ulceration and a flat elevation extended to the anal side along the fistula (red line).

**Fig. 5:**
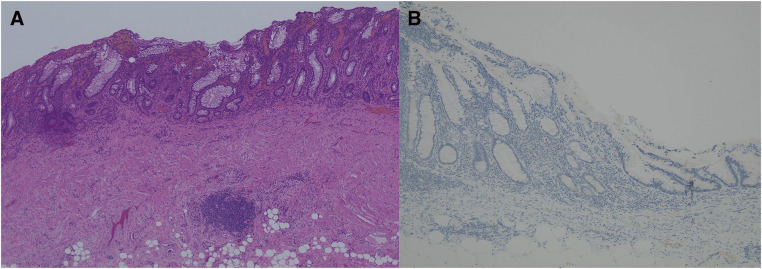
Histopathological examination. (**A**) Well-differentiated adenocarcinoma containing mucus in ileal mucosa with inflammatory changes. (**B**) The ileal mucosa and the entire tumor showed a complete absence of p53.

A subtotal circumferential mass was palpated on the lateral side of the anastomosis. His serum carcinoembryonic antigen was elevated to 18.01 ng/mL. Preoperative contrast-enhanced CT showed a 48-mm contrast-enhancing mass with a low-density region at the oral side of the ileoanal anastomosis in the ileal pouch and 4 enlarged lymph nodes in the mesentery of the ileum, but no metastases were detected (**Fig. 3A** and **3B**). Preoperative MRI showed tumor infiltration into the levator ani muscle. The preoperative diagnosis was ileal pouch cancer after restorative proctocolectomy with IPAA for UC, T4bN2M0 stage IIIB (UICC-TNM, 8th), and he underwent excision of the ileal pouch body, the ileoanal anastomosis, and creation of ileostomy. Partial prostatectomy and partial resection of the levator ani muscle was added to the operation due to suspicion of tumor infiltration into the prostate. Pathological examination showed mucinous carcinoma in the ileal mucosa with chronic inflammation and there was no immunostaining for p53 (**Figs. 4A** and **4B**, **5A** and **5B**). The tumor cells infiltrated into the subserosa; however, all lymph nodes were free of disease and his postoperative stage was pT3N0M0 stage IIA (UICC-TNM, 8th). No postoperative chemotherapy was administered, and at 6 months postoperatively, the patient remained recurrence free.

## DISCUSSION

Proctocolectomy with IPAA has become the standard surgical procedure for UC. Ileal pouch cancer is a rare but potentially fatal complication after proctocolectomy. “Pouch cancer” has been defined as cancer of the ATZ or ileal pouch mucosa in previously reported cases.

Most cases of pouch cancer were related to cancers arising from the ATZ after stapled IPAA, and case reports of cancer arising from ileal pouch mucosa are very rare. The cause of cancer arising from ileal pouch mucosa has been reported to be chronic inflammation in the ileal pouch, which can lead to dysplasia and adenocarcinoma. In cases of ileal pouch biopsy showing “type C inflammation” with atrophy of the ileal mucosa, it has been suggested that ileal mucosal inflammation is associated with ileal pouch cancer.^5–7)^ Atrophic changes in ileal mucosa associated with chronic inflammation, such as villous atrophy, are thought to contribute to carcinoma. In this case, histopathological examination showed no residual rectal mucosa, but tumor cells were detected in the ileal mucosa with inflammatory changes and villous atrophy. In addition, tumor cells showed a complete absence of p53. Based on the above findings, our case was related to inflammatory carcinogenesis. Although a fistulous ductal structure was present due to partial necrosis of the tumor and rupture of the muscularis, fistula cancer was excluded because no fistula was observed on pouchoscopy until the diagnosis of ileal pouch cancer.

The cumulative incidence of ATZ/ileal pouch cancer after ileal pouch surgery has been reported to be 0.9% at 5 years, 1.3% at 10 years, and 5.1% at 25 years postoperatively.^1)^ In a retrospective study of 3672 patients who underwent pouchoscopy after IPAA, the incidence of ileal pouch cancer after IPAA is very low, with a reported incidence of 0.016%.^8)^ A search on PubMed shows that 17 cases of ileal pouch adenocarcinoma, excluding those arising from ATZ, have been reported up to 2023,^5,9–22)^ composed of 13 female cases and 5 male cases. Including this case, the median age at the time of IPAA was 35 years (13–61 years), the median time from ileal pouch surgery to the diagnosis of ileal pouch cancer was 14 years (2–33 years), and the median age at the diagnosis of ileal pouch cancer was 50 years (24–75 years). Postoperative histopathological examination revealed 3 cases of well-differentiated adenocarcinoma (16.7%), 6 cases of moderately differentiated adenocarcinoma (33.3%), 5 cases of poorly differentiated adenocarcinoma (27.8%), and 2 cases of mucinous adenocarcinoma (11.1%).

Several risk factors for ileal pouch cancer have been reported. These include neoplasia when IPAA was performed,^23)^ more than 10 years of UC, complications of primary sclerosing cholangitis (PSC), and villous atrophy or chronic atrophic pouchitis of the ileal pouch mucosa.^24)^ A long history of backwash ileitis has also been reported as a risk factor for the development of dysplasia.^18)^ In the previous case reports, 16 out of 18 cases (88.9%) showed risk factors for the development of ileal pouch cancer, with ileal pouchitis being the most common, observed 14 cases (77.8%). Eight patients (44.4%) were diagnosed with dysplasia or adenocarcinoma on histopathological examination at the time of restorative proctocolectomy. In our case, postoperative histopathological examination showed villous atrophy of ileal pouch mucosa, but histopathological examination at the time of restorative proctocolectomy showed no neoplasia, no long-term backwash ileitis, and no complications of PSC. However, he developed ileal pouch cancer more than 30 years after the onset of UC. Having more than 10 years since developing UC was reported as a risk factor for ileal pouch cancer.^25)^ This case is considered a very rare ileal pouch adenocarcinoma with no risk factors other than ileal pouchitis and a long duration after developing UC.

In recent years, the number of patients with UC has been increasing in Japan, and the number of cases after ileal pouch surgery has also been rising. Cases of UC that undergo surgery at a young age require long-term postoperative surveillance, but there is currently no consensus on endoscopic surveillance after IPAA. The mortality rate of ileal pouch cancer at 2 years after diagnosis is reported to be 42.9%.^26)^ Early diagnosis of ileal pouch cancer by endoscopic surveillance is important because it has a poor prognosis. Japanese guidelines recommend endoscopic surveillance to observe the preserved anal canal after stapled IPAA, but there is no statement of follow-up after hand-sewn IPAA.^2)^ The European Crohn’s and Colitis Organisation guidelines recommend annual endoscopy after surgery for cancer, dysplasia, or in cases of PSC. However, there are no clear recommendations regarding postoperative follow-up for other conditions.^3)^ The American Society for Gastrotinestinal Endoscopy guidelines recommend annual endoscopy in high-risk cases of ileal pouch cancer with a history of dysplasia or cancer.^4)^ In addition, the guidelines state that annual surveillance may be considered in cases of refractory ileal pouchitis and ileal pouch mucosal atrophy associated with severe inflammation. In this case, ileal pouch cancer was diagnosed following endoscopy for hematochezia. The patient was not eligible for endoscopic surveillance according to existing guidelines, but more than 30 years had passed since he developed UC. In previous surveys, having more than 10 years since developing UC was identified as one of the risk factors of ileal pouch cancer.^25)^ In our study, the median time from the onset of UC to the development of ileal pouch cancer was 14 years,^5,9–22)^ which was consistent with the previous reports. Based on the above findings, it is considered desirable to include cases that have passed more than 10 years since the onset of UC in surveillance after ileal pouch surgery. Moreover, endoscopic surveillance is necessary to keep an eye on the development of ileal pouch cancer, even if the patient remains asymptomatic after IPAA, as in our case. Follow-up intervals should also be shortened in the presence of symptomatic or chronic inflammatory findings.

In our case, the biopsy, which was performed at the time of an endoscopy a year ago, was diagnosed as LGD, but a biopsy performed 1 year later at the time of an endoscopy for examination of hematochezia was diagnosed as adenocarcinoma. In cases of inflammatory carcinogenesis, the proliferative cell zone of the tumor is at the base of the grand,^27)^ and biopsy diagnosis may underestimate the degree of atypia. The possibility that submucosal cancer was present at the time of the initial biopsy cannot be ruled out because the second endoscopy performed for fecal occult blood showed an enlargement of the lesion. In addition, the postoperative histopathological examination showed that the tumor had extended into the submucosa like that of a fistula. There was an interval of about 1 year between the diagnosis of the second endoscopy for fecal occult blood and the endoscopy performed to examine blood in the stool. Japanese guidelines recommend endoscopic surveillance every 3 months for LGD with elevated lesions.^28)^ Since chronic inflammation of the ileocecal mucosa after IPAA occurs in about 6 months,^7,29)^ it is suggested that pouchoscopy should be performed 1 year after IPAA.^18)^ Endoscopic surveillance should begin about 6 months after IPAA, and shorter endoscopic examinations should be performed every 3–6 months in addition to annual surveillance if patients show symptoms or dysplasia is detected on biopsy at the annual endoscopic examination.

There is still no consensus on the management of dysplasia or cancer arising in the ileal pouch. For LGD, strict follow-up and endoscopic resection are considered. In contrast, high grade dysplasia and adenocarcinoma are difficult to be resected endoscopically. For this reason, resection of ileal pouch is recommended.^30)^ In previous case reports, surgery was performed in all 18 cases, including 8 cases of perineal ileocecal resection, 9 cases of ileal pouch resection, and 1 case of total pelvic visceral resection. In our case, preoperative imaging suggested invasion into the anorectal muscles and enlarged regional lymph nodes, and intraoperative findings suggested tumor infiltrating into the prostate gland. Consequently, partial resection of both the anorectal muscles and prostate was performed in addition to ileal pouch resection. Resection of the infiltrated organs or total pelvic exenteration may be required in addition to resection of ileal pouch body if preoperative imaging and intraoperative findings show tumor invasion into pelvic organs.

There are not many reports on the prognosis of ileal pouch cancer. The mortality rate for patients with ileal pouch adenocarcinoma is reported to be 30%, and 3 of 11 patients with ileal pouch cancer died within a year.^1)^ Of the 18 cases of ileal pouch adenocarcinoma searched on PubMed, 9 cases were stage II or higher. In the percentage of each histological type of ileal pouch cancer, poorly differentiated adenocarcinoma and mucinous adenocarcinoma were reported to account for 11.1% and 9.3% of all cases, respectively.^31)^ In this study, postoperative histopathological examination showed that the number of poorly differentiated adenocarcinoma and mucinous adenocarcinoma were 5 (27.8%) and 2 (11.1%) cases, respectively. It suggests that ileal pouch cancer may have an impact on prognosis. In our case, the biopsy performed 1 year later showed adenocarcinoma, whereas the biopsy performed a year ago showed LGD. Postoperative histopathological examination revealed well-differentiated mucinous adenocarcinoma pT3N0M0 stage IIA (UICC-TNM, 8th), but the patient is currently alive and recurrence free 6 months after surgery. Ileal pouch cancer has a poor prognosis; however, appropriate follow-up of dysplasia enables early detection of cancer prior to disease progression, and may improve the prognosis.

## CONCLUSION

Ileal pouch cancer is a rare complication of ileal pouch surgery for UC, but it can occur after a long period following surgery. The prognosis of ileal pouch cancer is generally poor. Therefore, endoscopic surveillance for detecting ileal pouch cancer is necessary for early detection and radical resection, especially if it occurs more than 10 years after the onset of UC.

## DECLARATIONS

### Use of artificial intelligence tools

We did not use any AI tools.

### Funding

No funding was received to assist with the preparation of this manuscript.

### Authors’ contributions

Writing—original draft preparation: Tetsuhiro Urashima.

Writing—review and editing: Tetsuhiro Urashima, Kenji Tatsumi, and Kazutaka Koganei.

Nao Obara, Eiichi Nakao, Sayumi Saito, Koki Goto, Hirosuke Kuroki, Kazutaka Koganei, and Akira Sugita provided critical advice for the preparation of this report.

All authors have read and agreed to the published version of the manuscript.

### Availability of data and materials

All data supporting the conclusions of this study are included in this published article.

### Ethics approval and consent to participate

This work does not require ethical considerations or approval.

### Consent for publication

Written informed consent was obtained from the patient for the publication of this case report and any accompanying images.

### Competing interests

The authors declare that they have no competing interests.
